# Recyclable Zr/Hf-Containing Acid-Base Bifunctional Catalysts for Hydrogen Transfer Upgrading of Biofuranics: A Review

**DOI:** 10.3389/fchem.2021.812331

**Published:** 2021-12-21

**Authors:** Yixuan Liu, Xixi Liu, Mingrui Li, Ye Meng, Jie Li, Zehui Zhang, Heng Zhang

**Affiliations:** ^1^ State Key Laboratory Breeding Base of Green Pesticide and Agricultural Bioengineering, Key Laboratory of Green Pesticide and Agricultural Bioengineering, Ministry of Education, State-Local Joint Laboratory for Comprehensive Utilization of Biomass, Center for Research and Development of Fine Chemicals, Guizhou University, Guiyang, China; ^2^ Key Laboratory of Catalysis and Materials Sciences of the Ministry of Education, South-Central University for Nationalities, Wuhan, China

**Keywords:** transfer hydrogenation, meerwein-ponndorf-verley reaction, Zr/Hf-containing catalyst, acid-base bifunctionality, biomass conversion

## Abstract

The massive burning of a large amount of fossil energy has caused a lot of serious environmental issues (e.g., air pollution and climate change), urging people to efficiently explore and valorize sustainable alternatives. Biomass is being deemed as the only organic carbon-containing renewable resource for the production of net-zero carbon emission fuels and fine chemicals. Regarding this, the selective transformation of high-oxygen biomass feedstocks by catalytic transfer hydrogenation (CTH) is a very promising strategy to realize the carbon cycle. Among them, the important Meerwein-Ponndorf-Verley (MPV) reaction is believed to be capable of replacing the traditional hydrogenation strategy which generally requires high-pressure H_2_ and precious metals, aiming to upgrade biomass into downstream biochemical products and fuels. Employing bifunctional heterogeneous catalysts with both acidic and basic sites is needed to catalyze the MPV reaction, which is the key point for domino/cascade reaction in one pot that can eliminate the relevant complicated separation/purification step. Zirconium (Zr) and hafnium (Hf), belonging to transition metals, rich in reserves, can demonstrate similar catalytic efficiency for MPV reaction as that of precious metals. This review introduced the application of recyclable heterogeneous non-noble Zr/Hf-containing catalysts with acid-base bifunctionality for CTH reaction using the safe liquid hydrogen donor. The corresponding catalysts were classified into different types including Zr/Hf-containing metal oxides, supported materials, zeolites, metal-organic frameworks, metal-organic hybrids, and their respective pros and cons were compared and discussed comprehensively. Emphasis was placed on evaluating the bifunctionality of catalytic material and the key role of the active site corresponding to the structure of the catalyst in the MPV reaction. Finally, a concise summary and prospect were also provided centering on the development and suggestion of Zr/Hf-containing acid-base bifunctional catalysts for CTH.

This review introduced the application of recyclable heterogeneous non-noble Zr/Hf-containing catalysts with acid-base bifunctionality for CTH reaction using the safe liquid hydrogen donor. The corresponding catalysts were classified into different types including Zr/Hf-containing metal oxides, supported materials, zeolites, metal organic frameworks, metal organic hybrids, and their respective pros and cons were compared and discussed comprehensively. Emphasis was placed on evaluating the bifunctionality of catalytic material and the key role of active site corresponding to the structure of the catalyst in the MPV reaction.

## Introduction

**SCHEME 1:**
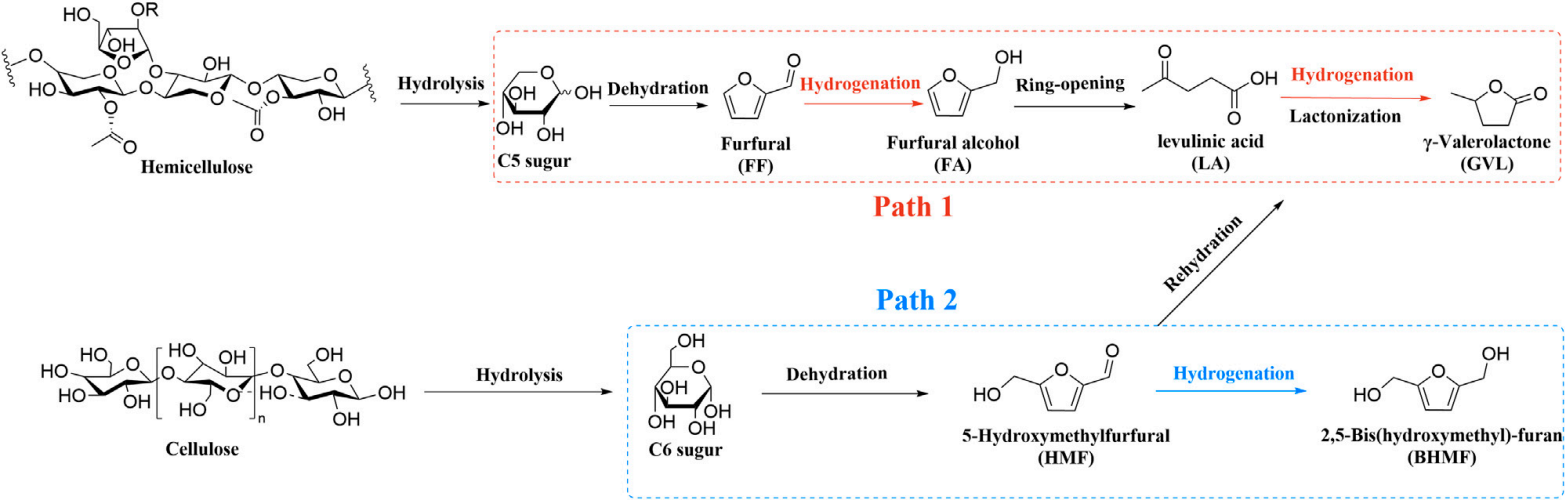
A common pathway for upgrading cellulose and hemicellulose.

The large-scale exploitation of fossil-based resources exists alongside environmental deterioration like global warming, acid rain, and air pollution (Tripathi et al., 2016; Valderrama Rios et al., 2018; Ahmad et al., 2019; Brauer et al., 2021; Schwarzman et al., 2021). On the eve of the “26th United Nations Climate Change Conference” in 2021, more than 230 major journals around the world jointly published an editorial to warn global leaders to act immediately for climate change (Atwoli et al., 2021). These serious problems compel humans to seek renewable and clean resources for the sustainable supply of fuels and chemicals (Zhang et al., 2020; Khan et al., 2021; Osman et al., 2021; Rode et al., 2021; Wu et al., 2021; Yu et al., 2021; Zantye et al., 2021; Zhang et al., 2021a). Biomass acts as the only organic carbon-containing source on earth that mainly contains carbon, hydrogen, and oxygen species (Wang et al., 2014b; Han et al., 2019; Spinelli et al., 2019; Zhu et al., 2019; Pattnaik et al., 2021). Biomass energy is ubiquitous, rich, renewable, and sustainable and its main sources are firewood, wood waste, agricultural straw, livestock manure, sugar crop waste, municipal waste with sewage, and aquatic plants, respectively (Li et al., 2018a; Luo et al., 2019; Abomohra et al., 2020; Sudarsanam et al., 2020; Wu et al., 2020). Compared with other renewable energy sources, it has unique advantages in terms of the reuse of waste resources and the production of fuels and high-value chemicals. It is highly valued in many aspects such as scientific research, politics, and the economy (Wu et al., 2018a; Mathimani and Mallick, 2019; Welch et al., 2021; Tan et al., 2022). There is a significant long-term two-way causal relationship between biomass energy consumption and economic growth (Ajmi and Inglesi-Lotz, 2020; Sabyrzhan et al., 2021). Hence, biomass has broad application potential in the future production and life of mankind (Kumar et al., 2015; Gawade et al., 2016; Hu et al., 2017; Liu et al., 2021c). However, compared to the desired products especially for fuels or fuel additives, biomass-derived feedstocks generally possess higher oxygen content (Prasomsri et al., 2013; Liao et al., 2014; Li et al., 2020a; Li et al., 2020b; Wang et al., 2020a). It is still necessary to further process the biomass feedstock to upgrade its functional groups (Dabros et al., 2018; Li et al., 2019b; Iglesias et al., 2020 Tan et al., 2021). In the field of biomass upgrading, the most widely used approach is to convert biomass (e.g., cellulose and hemicellulose) into a series of furan compounds *via* cascade reactions (Scheme 1) (Zhou and Zhang, 2016; Liu et al., 2019; Chen et al., 2021b). Among furan compounds, *γ*-valerolactone (GVL) is recognized as a kind of versatile chemical building block (Liu et al., 2021a; Kerkel et al., 2021; Delgado et al., 2022). It’s a green organic solvent and can also be used as a precursor for the manufacture of liquid fuels (Huang et al., 2018; Crabtree, 2019). 5-Hydroxymethylfurfural (HMF) has various furan rings coupled with the hydroxyl and aldehyde groups present at the exocyclic carbon atoms (Chen et al., 2018; Zhang et al., 2021b; Sarkar et al., 2021). It can be converted to levulinic acid (LA) and further produced GVL. or it can be converted to 2,5-bis(hydroxymethyl)furan (BHMF) through another route. (Kou et al., 2016; Priecel et al., 2018; Li and Jiang, 2019). Therefore, the main conversion path of this article is the production of furan compounds such as GVL and BHMF.

**GRAPHIC ABSTRACT:**
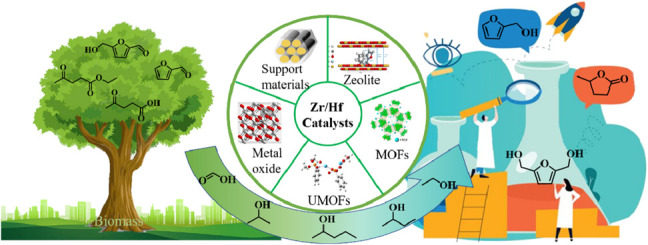


**FIGURE 1:**
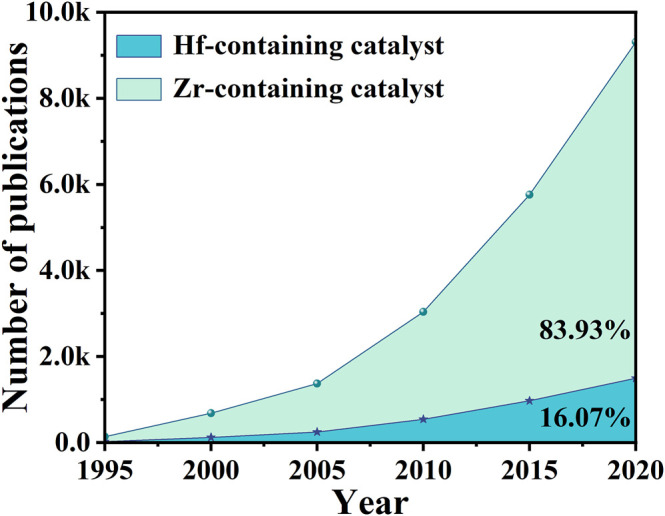
Comparison of the number of publications on Zr-containing catalysts and Hf-containing catalysts. Source: web of science (a search carried out in November 2021).

Many reactions are involved in these processes, such as dehydration, hydrogenation, ring-opening (Yang et al., 2017b; Iriondo et al., 2017; Solanki and Rode, 2019). But the key speed-determining step is transfer hydrogenation via the Meerwein-Ponndorf-Verley (MPV) reduction reaction, which is also called indirect catalytic transfer hydrogenation (CTH) (Singh et al., 2015; Li et al., 2018c; Shao et al., 2021). Different kinds of alcohols can be used as H-donors in relatively mild conditions, achieving efficient catalysis performance for CTH reaction of the carbonyl group of α, β-unsaturated aldehyde/ketone (Mondelli et al., 2020). Therefore, the C=O bond is hydrogenated while retaining the C=C bond (Wu et al., 2018b; Li et al., 2018d; Jin et al., 2019; Liu et al., 2021d). Heterogeneous catalysts were wildly used in MPV reduction reactions, which can be easily recycled and also can effectively reduce industrial production costs (Santana and Krische, 2021; Pan et al., 2022). It goes without saying that the choice of materials is of great importance for the relevant catalytic system. In contrast to the indirect hydrogenation via MPV reaction using alcohol as a hydrogen donor (H-donor), there is another direct hydrogenation reaction in CTH that uses precious metals and H_2_. Although precious metals (*e.g.*, Ru, Pd, Ir, Pt) were widely studied for direct CTH, the prices were not suitable for industrialization (Tuteja et al., 2014; Brethauer and Studer, 2015; Cao et al., 2017; Wang T. et al., 2020). On the other hand, non-noble metals (*e.g.*, Fe, Co, Cu, Ni) were restricted because of relatively low activity and/or product selectivity (Hu et al., 2019a; Zhang et al., 2019b; Shi et al., 2019; Jiang et al., 2020). These disadvantages have prompted scientists to explore alternative catalysts, which can balance economy and high selectivity (Yin and Shen, 2020). Thus, researchers discovered that transition metal carbides have similar properties with Pt metal (Pang et al., 2019). Since transition metals have both metallic and acidic properties, they can be used as effective catalysts for the upgrading of oxygen-containing compounds derived from biomass (Choi et al., 2011; Heard et al., 2016; Gonell et al., 2017; Wagner et al., 2021). It has good corrosion resistance and is not easily corroded by general acid-base aqueous solutions (Sullivan et al., 2016; Wei et al., 2021). Among the transition metals, Zr and Hf metals with similar properties have set off a wave of research craze in the field of CTH (Champness, 2011; Li et al., 2016a; B et al., 2018). Figure 1 shows that from 1995 to 2021, the number of articles published on Zr-based catalysts in 2020 is even 5 times than that of Hf-based catalysts. Although the metals of the fourth subgroup have similar properties, the research of Hf-based catalysts in this field is far from enough, which is also a trend and direction of future exploration.

There are some relevant reviews about biomass conversion and CTH reaction. For example, a comprehensive review of bifunctional catalysts to convert biomass into biofuels was proposed by Li et al. (2016a). What’s more, some reviews focused on the production of GVL from lignocellulosic biomass, (Tang et al., 2014b; Ye et al., 2020) and other studies paid attention to the mechanism and interface effects of heterogeneous CTH (Gilkey and Xu, 2016; Liu et al., 2021b). However, as far as we know, there is no review of the application of transition metal Zr/Hf catalysts used for the synthesis of biofuranics compounds. In this review, the target is to selectively discuss the latest developments of various types of Zr/Hf-containing catalysts through MPV reduction to upgrade biomass-derived feedstocks. It will focus on exploring the specific mechanism of the catalysts’ active site (Lewis/Brønsted acid and base) in each biomass upgrading reaction. Finally, some opportunities and challenges faced by CTH for efficiently converting biomass into fuels or high-value chemicals have also been prospected appropriately.

## Zr/Hf-Containing Metal Oxide Catalysts

Zr has excellent high-temperature resistance, and its melting point is as high as 2,700°C. Even if it is heated to 1900°C, it will still not react with molten Al, Fe, Ni, Pt, and other metals. Commercial zirconium oxide (ZrO_2_) has been used in the nuclear energy industry, national defense industry, electronic components, ceramic carbon powder, along with high-temperature resistant materials, etc. (Ishikawa et al., 2017). Among them, ZrO_2_ was also used in the field of catalysis due to its excellent chemical stability and high surface chemical activity (Liu et al., 2002; Zhu et al., 2004; Gonell et al., 2017). As a typical weak acid-base bifunctional material, ZrO_2_ and HfO_2_ have exceptional catalytic performance in CTH reaction (Paniagua et al., 2021). In Zr/Hf-containing metal oxide catalysts, as shown in Table 1, the main researches in recent years are classified and summarized according to the reaction substrate.

**TABLE 1:** Zr/Hf-based metal oxide catalysts.

Entry	Substrate	Catalyst	Condition	H-donor	Product	Conv. (%)	Yield (%)	References
1	LA/FA	Ag–Ni/ZrO_2_	220°C, 5 h	Water	GVL	100	99	Hengne et al. (2014)
2	LA	HCl/ZrO (OH)_2_	240°C, 2 h	2-BuOH	GVL	99.9	92.4	Tang et al. (2015)
3	LA	Cu–ZrO_2_	200°C, 5 h	Water	GVL	100	99.9	Hengne and Rode, (2012)
4	ML	Cu–ZrO_2_	200°C, 5 h	Methanol	GVL	95	92	Hengne and Rode, (2012)
5	ML	Ni/ZrO_2_	90°C, 20 h	2-PrOH	GVL	100	92	Sakakibara et al. (2019)
6	ML	Cu/ZrOCO_3_	180°C, 7 h	2-PrOH	GVL	99	89.79	Ma et al. (2020)
7	EL	ZrO(OH)_2_·*x*H_2_O	240°C, 1 h	EtOH	GVL	89.1	75.3	Tang et al. (2014a)
8	EL	ZrO_2_-B_2_O_3_	200°C, 4 h	2-PrOH	GVL	95.1	88.5	He et al. (2016a)
9	EL	ZrO_2_	250°C, 3 h	EtOH	GVL	95.5	81.5	Tang et al. (2013)
10	EL	Al_7_Zr_3_-300	220°C, 4 h	2-PrOH	GVL	95.5	83.2	He et al. (2016b)
11	EL	ZrFeO_x_	270°C, 3 h	EtOH	GVL	94.2	87.2	Li et al. (2015)
12	EL	Zr_5_Ni_5_	200°C, 3 h	2-PrOH	GVL	97.2	95.2	Li et al. (2016b)
13	EL	Ti/Zr	180°C, 6 h	2-PrOH	GVL	100	90.1	Yang T. et al. (2017)
14	BL	ZrO_2_	150°C, 16 h	2-BuOH	GVL	99.9	84.7	Chia and Dumesic (2011)
15	FF	Zr(OH)_4_@CoFe_2_O_4_	160°C, 4 h	2-PrOH	FA	95.4	92.6	Hou et al. (2021)
16	FF	HfO(OH)_2_·*x*H_2_O	180°C, 8 h	2-PrOH	GVL	100	64.2	Li M. et al. (2021)
17	HMF	ZrO(OH)_2_	150°C, 2.5 h	EtOH	BHMF	94.1	88.9	Hao et al. (2016)

### LA and its Esters as Substrates

#### LA as a Substrate

**SCHEME 2:**
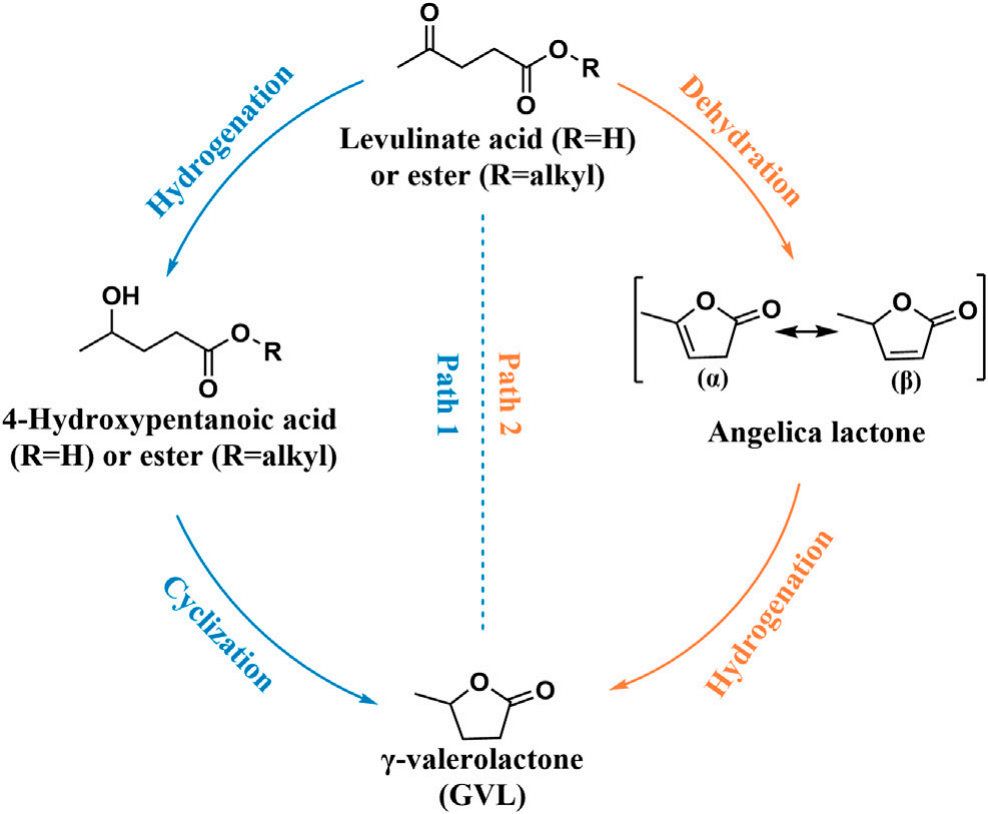
Two pathways for the conversion of LA and its esters to GVL.

LA was listed as one of the 12 high-value chemicals from biomass by the U.S. DE (Bogale et al., 2019). It was considered as a significant platform compound for the synthesis of organic chemicals (Hu et al., 2021; Tian et al., 2021). In general, there are usually two reaction pathways of heterogeneous catalysis in the production of GVL using LA and its esters [e.g., methyl levulinate (ML), ethyl levulinate (EL), butyl levulinate (BL)] as substrates. These two reaction pathways mainly depend on the controlled reaction conditions and the catalyst used during the reaction (Scheme 2) (Dutta et al., 2019). In path 1, hydrogenation reaction is prone to occur at lower temperatures to promote the conversion of LA to 4-hydroxyvaleric acid under the water phase system, which is then dehydrated to GVL under the action of a catalyst. In path 2, LA is first dehydrated to form angelica lactone at higher temperatures, which is then hydrogenated to obtain GVL. In short, hydrogen and dehydration can occur either way. But under different control reaction conditions, the sequence of these two processes will change, accordingly.

Ag–Ni/ZrO_2_ catalyst has attracted researchers’ attention due to its magnetic properties that are easy to separate (Hengne et al., 2014). The two metals play a synergistic effect in the transformation of LA and furfuryl alcohol (FA) (1:1) mixture to GVL. It also proved that Ag–Ni/ZrO_2_ has a comprehensive scope of applications in the one-pot CTH of biomass-derived C3 to C6 molecules, with relatively high conversion and selectivity (>80%) (entry 1 of Table 1). In addition to using high-purity synthetic chemicals as substrates, researchers were further required to explore some methods that can be closer to biomass raw materials to produce GVL. Tang et al. developed an *in-situ* catalyst generation system to produce GVL (Tang et al., 2015). The system can decompose the HCl/ZrO(OH)_2_ catalyst in LA and 2-butanol (2-BuOH) solution autonomously and then a 2-BuOH *in-situ* H-donor was employed to catalyze the cyclization of LA esterification to GVL. The highlight of this system was that crude LA can be directly used to produce GVL. The crude LA was extracted from the acid hydrolysis of cellulose (47.8% yield), and flowed into 2-BuOH. Finally, 2-BuOH was used to extract LA and FA from the cellulose hydrolysate (90.2% v. s. 15.1%), as depicted in entry 2 of Table 1. Even in the presence of humin after use, a GVL yield of 82.0% can be successfully obtained. In addition, when investigating the influence of solvent, it was found that the GVL yield of 2-BuOH was much lower than that of 2-Propanol (2-PrOH) (27.5% vs. 62%) at 200°C because the steric effect of 2-BuOH was stronger than 2-PrOH. Continue to increase the temperature to 240°C, the steric effect of 2-BuOH can be overcome, and the yield can be increased to 84.5%. According to Derjaguin–Landau–Verwey–Overbeek (DLVO) theory, the principle of the catalysts agglomeration phenomenon was speculated. That was, the absorbed LA and H-donors enhanced the electrostatic repulsion between the ZrO(OH)_2_ particles formed *in situ*, thereby preventing further agglomeration in 2-BuOH. In other words, the dispersibility and morphology of ZrO(OH)_2_ particles that played a key catalytic role mainly depended on the solvent and the substrate. Most Cu-ZrO_2_ catalysts were prepared by co-precipitation with mixed precursors (entry 3 of Table 1) (Hengne and Rode, 2012). Its excellent catalytic performance should be attributed to its strong surface acidity. The effective active component in the catalytic hydrogenation process was the reduced Cu particles dispersed on the catalyst surface. The introduction of Cu into the tetragonal ZrO_2_ lattice can enhance the adhesion of these particles as well as make them dispersed. It can also enhance the number of acid sites, among which Lewis acid was also determined to enhance the transfer hydrogenation activity. Changing the starting material from LA to ML and the H-donor from water to methanol, Cu-ZrO_2_ still had a very objective activity (entry 4 of Table 1).

#### ML as a Substrate

Ni/ZrO_2_ was also used to convert ML and LA to GVL, which were catalyzed and reacted to test the active materials (Sakakibara et al., 2019). It was concluded that the Ni contributed to the hydrogenation of ML and LA, and the Zr contributed to the lactonization of the hydrogenated product. Under relatively mild conditions of 90°C, the GVL yield reached 92% (entry 5 of Table 1). But the disadvantage was that the reaction time was as long as 20 h. It is well known that GVL can be directly hydrogen transfer catalyzed by ZrOCO_3_. Further studies by Ma et al. found that using ZrOCO_3_ as a carrier, the introduction of Cu/Cu^+^ can increase the CTH activity (entry 6 of Table 1) (Ma et al., 2020). And Cu/ZrOCO_3_ can also further convert GVL into 1,4-Pentanediol (1,4-PDO) through the MPV reaction. This may be caused by the coordination effect between the Cu and the acid-base sites of the carrier. Cu vacancy (Cu^0^) and Cu ion (Cu^+^) were formed after *in-situ* reduction. Cu^+^ can be used as Lewis acid to adsorb and activate the C=O group through the electron lone oxygen atom pair. Alcohol can be excited to become an activated metal hydride on the Cu^0^ surface, and then attack the activated C=O group to form an O-H group. ZrOCO_3_ was an amphoteric catalyst, which can also promote the MPV reaction through six-membered ring intermediates. In addition, amphoteric carriers (such as Al(OH)_3_ and ZrOCO_3_) contained more acid-base sites than basic carriers (such as Mg_2_(OH)_2_CO_3_ and ferric hydroxide), demonstrating a better catalytic activity accordingly.

#### EL as a Substrate

EL was used to prepare GVL through CTH. Tang et al. used ZrO(OH)_2_ *x*H_2_O catalyst to obtain 89.1% EL conversion and 75.3% GVL yield in ethanol (EtOH) under 240°C for 1 h (entry 7 of Table 1) (Tang et al., 2014a). First, EtOH was adsorbed on the surface of the catalyst and dissociated into the corresponding alkoxide. EL obtained two hydrogen atoms from EtOH to obtain the intermediate ethyl 4-hydroxypentanoate (4-HPE). Then 4-HPE underwent intramolecular transesterification to obtain the final product GVL. But there was another reaction path, 4-HPE and EtOH were etherified to produce by-product 4-ethoxypentanoate (4-EPE). Self-aldol condensation may occur between EL, GVL, and aldehyde dehydrogenated from EtOH, and the formation of trace by-products can be ignored in the system. Doping boric acid in Zr-containing catalysts can increase its acid content, but the traditional wet impregnation method was difficult to control the leaching of the boron component during the reaction. Therefore, He et al. used the sol-gel method to improve the synthesis of the mesoporous Zr/boron mixed oxides catalyst (He et al., 2016a). ZrOCl_2_ 8H_2_O and boric acid were stirred at 40°C for 3 h and then transferred to an oven to evaporate the solvent for 4 h after sonication. After drying at 80°C for 1 day, it was calcined at 450°C for 6 h. In general, the preparation process was relatively simple. With this amorphous material catalyst, 95.1% EL conversion and 88.5% GVL yield were obtained at 200°C for 4 h (entry 8 of Table 1). As the boron content increased, the surface area, acid density, and alkali density of the mesoporous material improved correspondingly. Nonetheless, adding too much boron to ZrO_2_ will reduce the alkali density and inhibit the alkalinity of the catalyst. However, ZrO_2_ catalyst still existed the disadvantage of high temperature or long time during the reaction (entry 9 of Table 1) (Tang et al., 2013). To address this issue, Al-Zr mixed metal catalysts were prepared by the co-precipitation method (He et al., 2016b). ZrOCl_2_ 8H_2_O and Al(NO_3_)_3_ 9H_2_O were dissolved in deionized water, and then an aqueous ammonia solution (25–28%) was added dropwise. Next, the solution underwent a series of methods such as pH adjustment, aging, drying, and calcination to obtain the target solid catalyst. It was found that when Al was added to ZrO_2_, the surface area was enlarged and the number of effective acid and base sites of the catalyst was increased. With 2-PrOH as the H-donor and solvent, the EL conversion was 95.5% and the GVL yield was 83.2% at 220°C (entry 11 of Table 1). Both formic acid and LA can be obtained from sugars in an equimolar ratio were found by Li et al. Therefore, if FA is used as an H-donor and LA is used as a substrate to synthesize GVL, it is obviously a reasonable system. Nano-FeZrO_X_ materials have also been reported to have magnetic recyclable properties. The acid-base bifunctional catalysts of superparamagnetism were synthesized through the reaction of solvent heat treatment and hydrolysis condensation. Upon using EtOH as H-donor, effective conversion of EL to GVL was successfully conducted. A series of FeZrO_x_ catalysts with different Fe-Zr ratios were prepared (entry 11 of Table 1) (Li et al., 2015). It was found that ZrFeO (1:3)-300 with Fe_3_O_4_ covered by a ZrO_2_ layer had an appropriate distribution of acid-base sites as well as a medium surface area and pore size. The yield of GVL can be reached as high as 87.2% (3 h, 230°C). In addition, the catalyst containing Zr and Fe can also be combined with the solid acid HY2.6 to directly convert sugar into GVL, with a yield of 44.7%. To be specific, Ni(NO_3_)_2_ 6H_2_O and ZrOCl_2_ 8H_2_O were used to synthesize magnetic Ni-Zr nanocatalysts for the conversion of various biomass derivatives (Li et al., 2016b). This hydrogen-reduced magnetic Zr_5_Ni_5_ nanoparticles (<20 nm) catalyzed EL to produce GVL (95.2% yield) within 3 h at 200°C (entry 12 in Table 1). Surprisingly, the system can also catalyze the conversion of fructose, glucose, cellobiose, and carboxymethyl cellulose into GVL and EL in one pot, with a total yeild of 69.5, 60.1, 56.0, and 51.5%, respectively. And ICP-OES detected that only 0.3 wt% Ni and 0.5% Zr were immersed in 2-PrOH. The very small amount of immersion can clearly explain the heterogeneity of the catalyst and the nature of easy recycling. In addition, the nano-catalyst was combined with the solid acid HY6, the catalyst was easily attracted by the permanent magnet because of its magnetic properties. The used catalyst was washed 3 times with EtOH and dried, and it can be reused more than 5 times. In addition, the catalyst can be recovered by more than 89–93%, which is very suitable from an economic and convenient point of view.

Also using EL as a substrate, Yang et al. developed a microsphere Ti/Zr porous oxide catalyst through a sol-gel process combined with solvent heat treatment (Yang et al., 2017a). With hexadecyl amine (HDA) as the directing agent, the catalysts (TixZry) with different Ti/Zr molar ratios that have −Zr−O−O−Zr− network were developed. This not only allowed the mixed oxide to have adjustable porosity and large surface area but also enhanced its acidity and alkalinity. Compared to the amorphous structure of commercial titanium oxide (TiO_2_) and ZrO_2_, Ti/Zr oxides were spherical. Experiments and tests showed that Ti_2_Zr_8_ had the largest surface area (385 m^2^/g), appropriate acidity (1.12 mmol/g), and alkalinity (0.46 mmol/g), which was the best option. In general, if there was a base in the reaction system, the reaction activity will be improved. The increase in reactivity benefited from the ability to accept hydroxyl protons from the H-donor. There were also many sources of bases, which can be organic ligands, solvent molecules, or dissolved alkaline substances. The catalyst can be reused at least 6 times, and it can still obtain almost complete EL conversion and GVL yield of greater than 84.4% (entry 13 of Table 1). BL was also one of the common LA and its esters (entry 14 of Table 1) (Chia and Dumesic, 2011).

### FF (Furfural) as a Substrate

For the conversion of FF to FA, the hollow core-shell magnetic catalyst named Zr(OH)_4_@CoFe_2_O_4_, was developed and employed for CTH (entry 15 of Table 1) (Hou et al., 2021). Firstly, 2-PrOH was adsorbed on the surface of Zr(OH)_4_ and interacted with Lewis acid sites (Zr^4+^) and base sites (O^2-^) to form metal alkoxide. Then, the O atom on the carbonyl group of FF was activated by Zr^4+^. A typical six-membered ring transition state was formed. Then hydride transfer from alkoxide to the carbonyl group of FF was accomplished. Finally, the newly formed FA was desorbed from the surface of the catalyst along with acetone, and the active site of Zr^4+^ was re-exposed for the next run.

One-pot conversion of FF to GVL can use commercial HfCl_4_ as a catalyst (Li et al., 2021b). With 2-PrOH as H-donor, the yield of GVL reached 65.5% in 8 h at 180°C (entry 16 of Table 1). During the reaction, HfCl_4_ is hydrolyzed *in situ* to produce HfO(OH)_2_ *x*H_2_O (medium Lewis basic) and HCl (strong Brønsted acid). Together with the Lewis acid site (Hf^4+^), they play a synergistic effect in the cascade reaction process, which is significantly improved the catalytic activity. HfCl_4_ provides Lewis acid site (Hf^4+^), while HfO(OH)_2_·*x*H_2_O and HCl were gradually *in situ* generated by the HfCl_4_ hydrolysis in 2-PrOH due to the presence of residual water to provide Lewis base site (O^2-^) and Brønsted acid site (HCl), respectively. 1) The carbonyl group of FF was adsorbed on the Lewis acid site (Hf^4+^), and 2-PrOH with the oxygen and hydrogen atom of the hydroxyl group were respectively adsorbed to the Lewis acidic Hf^4+^ and the Lewis basic oxo-ion site. Then, a six-membered ring transition state was formed to complete the transfer hydrogenation process. FF was converted to Furfuryl Alcohol (FA), at the same time the 2-PrOH was transformed to acetone. 2) FA reacting with 2-PrOH was catalyzed by Lewis acid sites to form isopropyl furfuryl ether (FE) by etherification reaction. 3) The conversion of IPL to isopropyl 4-hydroxyvalerate (4-HPE) via transfer hydrogenation was similar to that of FF to FA, which was also catalyzed by the Lewis acid-base site (Hf^4+^-O^2-^). 4) 4-HPE undergone cyclization reaction to produce equivalent GVL was conducted in the presence of acid sites. In addition, the recycled catalysts can effectively catalyze the conversion of FF to FA after being calcined (2-PrOH as H-donor and the yield is 60.5% at 170°C for 1.5 h).

### HMF as a Substrate

**SCHEME 3:**
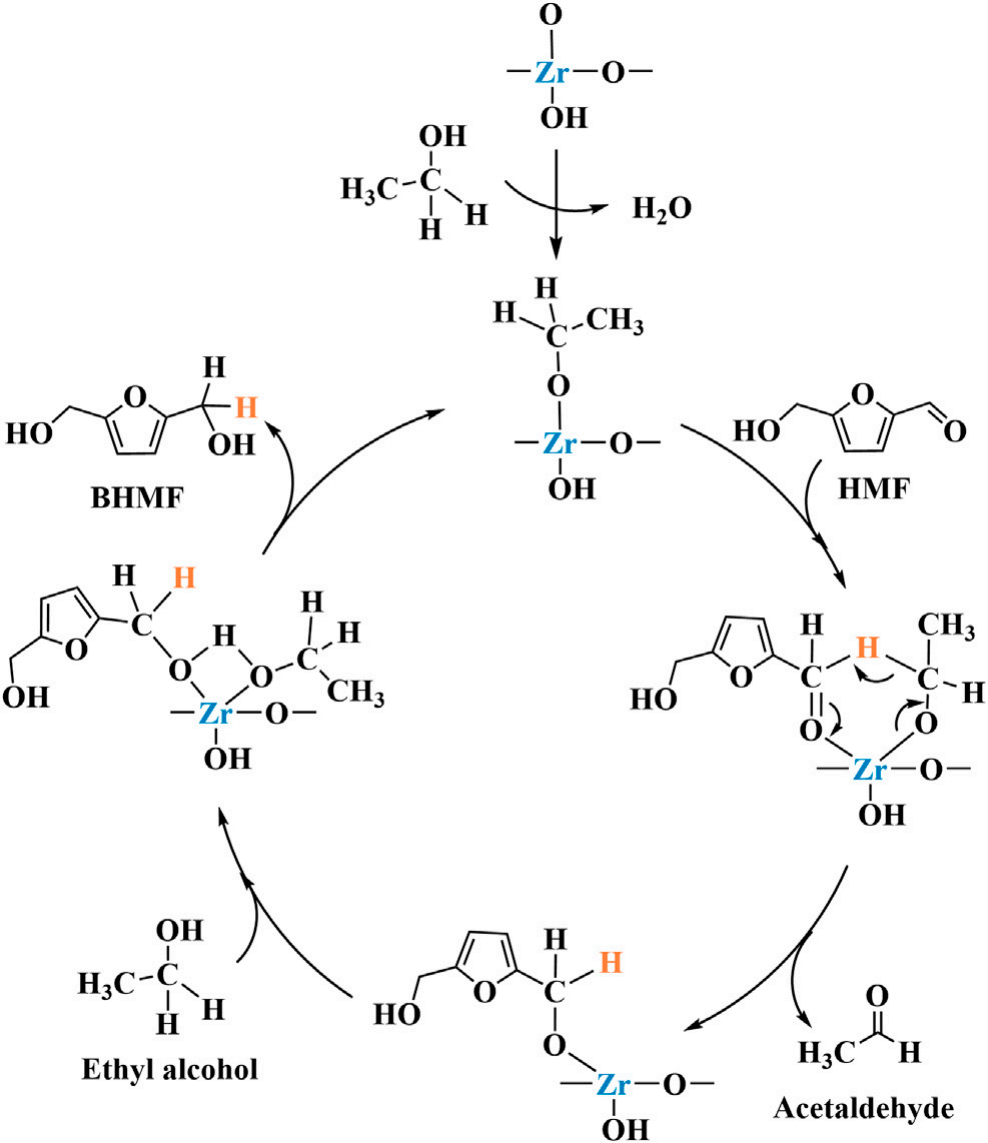
The mechanism for the CTH of HMF into BHMF over ZrO(OH)_2_ (Hao et al., 2016).

CTH mechanism of HMF on ZrO(OH)_2_ was shown in Scheme 3 (Hao et al., 2016). The carbonyl oxygen of HMF coordinated with the alkoxide a to form a six-membered ring transition state b on Zr species. The hydride was transferred from the alkoxide to the carbonyl of HMF in b. Meanwhile, the new carbonyl (aldehyde) dissociated and released the intermediate c. Then another EtOH coordinated with it to form d. At the end, the newly reduced carbonyl (BHMF) dissociated to regenerate the alkoxide a. The BHMF yield of 88.9% can be achieved by reacting for 2.5 h at 150°C (entry 17 of Table 1).

**SCHEME 4:**
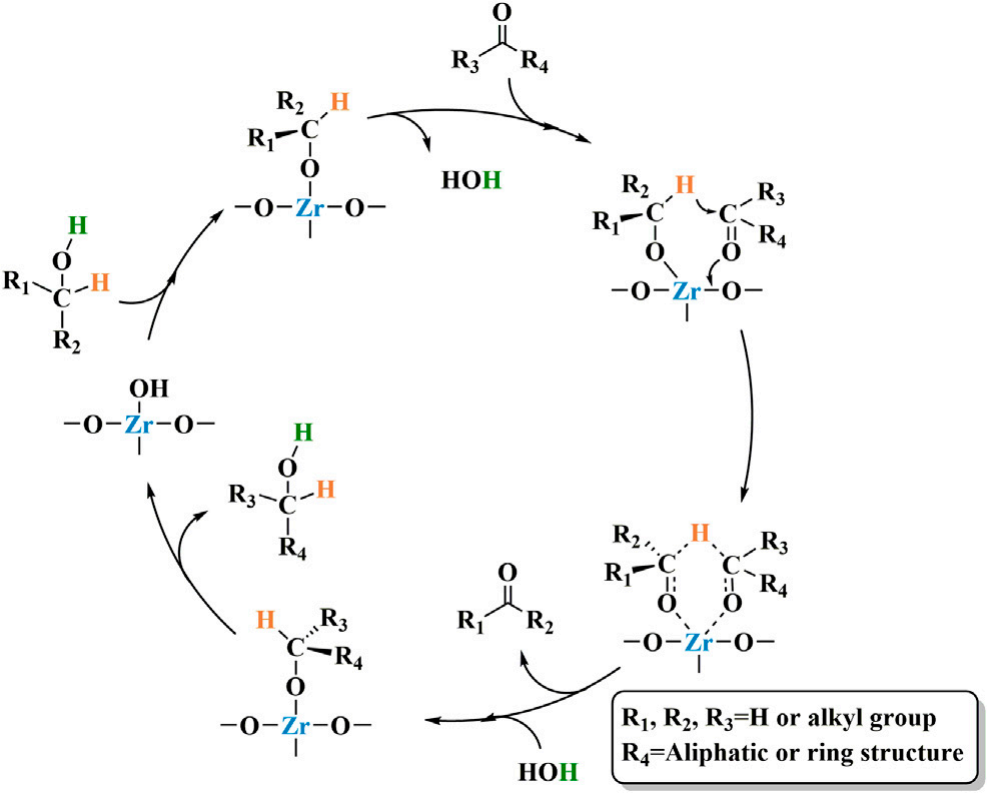
General reaction mechanism of hydrated ZrO_2_.

The mechanism by which Zr-containing metal oxides catalyze the completion of transfer hydrogenation of carbonyl compounds via MPV reduction reaction is shown in Scheme 4. First, the alcohol is absorbed by the Lewis acid sites in the catalyst to form the corresponding alkoxide (Gonell et al., 2017). Then, the carbonyl group combines with the alkoxide to produce a six-membered ring transition state (Injongkol et al., 2017). Finally, a coordinated hydrogen transfer occurs between the activated carbonyl compound and the alkoxide. process. At the same time, the obtained new carbonyl chemical substance ketone is released. Finally, another alcohol molecule participates in the reaction to generate the target product and the starting alkoxide.

## Zr/Hf-Containing Supported Material Catalysts

The carrier material usually uses mesoporous molecular sieves, which generally have relatively large specific surface areas, large pore sizes, along with regular pore structures (Taniya et al., 2010). Mesoporous molecular sieves are regarded as good shape-selective catalysts and have better catalytic activity and selectivity than zeolite molecular sieves when organic macromolecular substances participate (Shen et al., 2014). In addition, the pore size can be continuously adjusted in the range of 2–50 nm, and the pores and the large and silan-rich surface (up to 2000 m^2^/g) are easy to modify (Gao et al., 2021a). Mesoporous molecular sieves are often used as carriers to load or dope transition metal (e.g., Zr and Hf) elements and rare earth elements into their frameworks, surfaces, or pores. There is no doubt that mesoporous materials have also shown their talents in the field of biomass catalytic upgrading (Table 2).

**TABLE 2:** Zr/Hf-containing supported material catalysts.

Entry	Substrate	Catalyst	Condition	H-donor	Product	Conv. (%)	Yield	References
1	LA	ZrO_2_/SBA-15	150°C, 2 h	2-PrOH	GVL	99	83	Kuwahara et al. (2014)
2	ML	ZrO_2_/SBA-15	150°C, 2 h	2-PrOH	GVL	98	88	Kuwahara et al. (2014)
3	ML	ZrO_2_(10)/SBA-15	150°C, 3 h	2-PrOH	GVL	99.5	91	Kuwahara et al. (2017)
4	EL	ZrO_2_/SBA-15	150°C, 2 h	2-PrOH	GVL	86	81	Kuwahara et al. (2014)
5	EL	Chitosan–Hf	160°C, 8 h	2-PrOH	GVL	100	97	Wang T. et al. (2020)
6	EL	Chitosan–Zr	160°C, 8 h	2-PrOH	GVL	100	97	Wang T. et al. (2020)
7	FF	ZrO_2_-SBA-15	170°C, 7 h	2-PrOH	GVL	99	37	Iglesias et al. (2018)
8	FF	Fe_3_O_4_/ZrO_2_@MCM-41	150°C, 24 h	2-PrOH	GVL	99.3	80.8	Gao et al. (2021)
9	FF	TPA-ZrO_2_-SBA-15	170°C, 11 h	2-PrOH	GVL	100	81	Srinivasa Rao et al. (2021)
10	FF	Zr-SBA-15	110°C, 6 h	2-PrOH	FA	63	40	Iglesias et al. (2015)

### LA and its Esters as Substrates

Using LA as a reaction substrate, ZrO_2_ was supported on typical hexagonal mesoporous Si, such as Mobil Composition of Matter-41 (MCM-41) and Santa Barbara Amorphous-15 (SBA-15) (entry 1, 2, 4 in Table 2) prepared by Kuwahara et al. (2014). The silicon carrier itself is inactive, and the reaction kinetics demonstrated that the pore size of mesoporous Si had little effect on the catalytic activity. However, it was possible to increase the CTH reaction activity by expanding the surface area of the oxide support carrier, which can change the local structure of the Zr species. Specifically, the high surface area of Si support provided a surface environment that was suitable for the introduction of highly dispersed low-coordination Zr. The loss of used catalytic activity was due to the strong adsorption of organic residues on the catalyst surface. This prevented the next batch of organic substrates from entering the Zr active site. Similar catalysts have also been reported, and the conversion and yield have been further improved (entry 3 of Table 2) (Kuwahara et al., 2017). For the conversion of EL to GVL, using biopolymer chitosan as a support to prepare the efficient Zr-containing catalyst (chitosan–Zr) was developed by Wang et al. (2020c). Chitosan is derived from the diacylation of chitin (Synowiecki and Al-Khateeb, 2003). The world’s marine ecosystems produce about 1,600 million tons of chitin each year (Aydın and Aksoy, 2009). And it was the second kind of abundant biopolymer in nature after cellulose, but they were underutilized. Many–NH2 and–OH groups exist in the chitosan, which means it has a strong chelating ability for M^+^. The conversion rate of the relevant catalysts containing Zr or Hf can reach 100%, and the corresponding yield was 97% (entries 5 and 6 of Table 2). Undoubtedly, it was a wise strategy to use biomass-derived chemicals to prepare catalysts for the efficient valorization of biomass feedstocks.

### FF as a Substrate

Using FF as the starting substrate, the same material had been studied with different Zr coatings of ZrO_2_ using wet chemical methods (entry 7 of Table 2) (Iglesias et al., 2018). The first type was related to the strong acid position on the interface with Si. Another type of highly dispersed t-ZrO_2_ crystals that were ubiquitous in the second and third layers was related to the position of the weak acid. This catalyst was used for the cascade reaction of FF to GVL. In addition, pyridine titration experiments during vacuum and 2-PrOH soaking were also carried out, proving that the Lewis acidity of all ZrO_2_ monolayer thicknesses dominated the key role. A single ZrO_2_ monolayer had a few Brønsted acid sites. And this may be related to residual surface silanol. The etherification and isomerization of FA to EL reduced the reaction rate. Fe_3_O_4_/ZrO_2_@MCM-41 was tailored by the impregnation of ZrO_2_ on mesoporous MCM-41 support coated by Fe_3_O_4_. It could efficiently catalyze the cascade reaction in terms of conversion of FF to GVL (entry 8 of Table 2) (Gao et al., 2021). The introduction of these two substances did not change the original ordered hexagonal framework of MCM-41. The incorporation of Fe_3_O_4_ was a simple way to adjust its acidity. The kinetic study demonstrated higher activation energy for the LA-to-GVL step (86.9 kJ/mol) than that of FF transfer hydrogenation (35.0 kJ/mol) and that of subsequent alcoholysis process (51.0 kJ/mol). The catalyst could be fully recovered and reused because of its excellent magnetic property. By Zr K-edge XAFS analysis, it can be seen that the Zr^4+^ oxide species anchored on the silica surface in a low-coordination state played the most important role in the reaction. Another application of this catalyst was the conversion of FF and 2-methylfuran (2-MF) to diesel precursors through the hydroxyalkylation/alkylation (HAA) reaction (Luo et al., 2018). The mesoporous catalyst was prepared by using ZrO_2_ and phosphotungstic acid (TPA) on the inside and outside of the pores of SBA-15 (Srinivasa Rao et al., 2018) (entry 9 of Table 2). It has also been reported that Zr-SBA-15 performs average in transforming FF to FA (Iglesias et al., 2015). However, it has excellent performance when reducing cyclic ketones with secondary alcohols. After the reaction, the corresponding alcohol yield was as high as 99% (entry 10 of Table 2).

## Zr/Hf-Containing Zeolite Catalysts

Zeolite is a kind of nanoporous aluminosilicate crystal (Chai et al., 2021; Mardiana et al., 2022). Because of its regular pore structure and adjustable acidity, it is widely used in adsorption, separation, and catalysis (Sushkevich et al., 2014; Koreniuk et al., 2015; Wang et al., 2016c; Kim et al., 2020). The support material mainly uses mesoporous molecular sieves, which are used to provide templates to increase the stability and surface area of the catalyst, while zeolite will coordinate with the metal and form new bonds. As we all know, the catalytic activity of a molecular sieve is closely related to its unique acidity (Ramanathan et al., 2008; Melero et al., 2018; Cho et al., 2020). Usually, the acidity of zeolite is mostly affected by the isostructural substitution of Al^3+^ by Si^4+^ in the framework. This can create an imbalance of negative charges in the crystal lattice, and the concomitant exchangeable cations can make up for this imbalance (such as H^+^, Na^+^, K^+^). When protons are connected to oxygen atoms that are connected to silicon and Al atoms, the local charge transfer weakens and lengthens the O-H bond. In turn, this enhances the strength of its Brønsted acidity (Melero et al., 2017). The final result of these bonding interactions is that the local environment determines the type and intensity distribution of acidic sites. After a long-term research, scientists have found that if the concentration of substituted tetrahedral atoms does not exceed a certain threshold, Brønsted acid sites can show a constant acid strength which is suitable for adsorption and catalysis (Hernández et al., 2016). Therefore, the high intrinsic catalytic activity of zeolite is mainly derived from the excellent stability of the transition state in the nanopore confinement. Table 3 compares the catalytic effects of several different Zr/Hf-containing zeolite catalysts with the substrate as the classification standard.

**TABLE 3:** Zr/Hf-containing zeolite catalysts.

Entry	Substrate	Catalyst	Condition	H-donor	Product	Conv. (%)	Yield (%)	References
1	LA	Zr-Beta/Al-MFI-ns	120°C, 11 h	2-BuOH	GVL	100	96	Bui et al. (2013)
2	ML	Hf-Beta	160°C, -	2-BuOH	GVL	80	76.8	Luo et al. (2014)
3	EL	Zr-beat-100	150°C, 10 h	2-PrOH	GVL	100	96	Wang J. et al. (2014)
4	BL	(Zr)SSIE-beta	230°C, 24 h	2-BuOH	GVL	85	76	Antunes et al. (2016)
5	FF	Zr/Al-β-TUD1	120°C, -	2-BuOH	GVL	85	76	Antunes et al. (2016)
6	FF	Zr-HY/Al-HY	120°C, 5 h	2-PrOH	GVL	95	85	Zhang H. et al. (2019)
7	FF	Zr-Al-Beta	120°C, 3 h	2-PrOH	FA	99.6	97.3	Gao et al. (2020)

### LA and its Esters as Substrates

The reaction system of converting LA to GVL can use Zr-Beta and MFI topological structure nano-layer aluminosilicate (Al-MFI-ns) as the bicatalyst (entry 1 of Table 3) (Bui et al., 2013). Both of these steps resulted in the oxidation of the H-donor, which can be separated from the product. And it can be regenerated under mild gas-phase conditions and was directly put into use next time. It was noteworthy that this process can be achieved with cheap catalysts (e.g., Ni or Cu). Moreover, the combined catalysts can also convert hemicellulose to GVL. This also provides a new potential process for the transformation of pentoses to GVL.

The process of reducing ML to GVL by Lewis acid catalyst based on relevant kinetic parameters was described Luo et al. (2014). The reaction sequence and the rate of the mechanism were calculated. And the kinetic parameters of Ti, Sn, Zr, and Hf β-catalysts at different temperatures were analyzed (entry 2 of Table 3). Although the selectivity of GVL was over 94%, the turnover rate of these three zeolite catalysts was different during the MPV reaction process, and the Hf β catalyst had the highest activity. It is confirmed that the stronger the Lewis acid of the catalyst is, the more stable the six-membered ring transition state of the rate-limiting CTH step can be achieved (Assary et al., 2013). Kinetic studies were carried out by changing the H-donor (primary alcohol and secondary alcohol), proving that reducing the chain length of the alcohol did not affect the Ea.

EL was catalytically reduced to GVL by Zr-Beta zeolite. A small amount of Zr (Si/Zr ∼75–200) was incorporated into the zeolite (Wang et al. (2014a). The Zr-Beta zeolite catalyst with isolated Zr atoms with Lewis acidity was prepared (entry 3 of Table 3). Density functional theory (DFT) calculation studies showed that Zr^4+^ was located in unique crystallographic positions of zeolite. To test its industrialization potential, the catalyst effect was further tested in the batch reactors and continuous flow reactors. Through the investigation of the mechanism, it was found that Brønsted acid sites can cause other side reactions. For example, the production of hemiacetals, pentenes, and their isomers reduced the selectivity to GVL.

### FF as a Substrate

**FIGURE 2:**
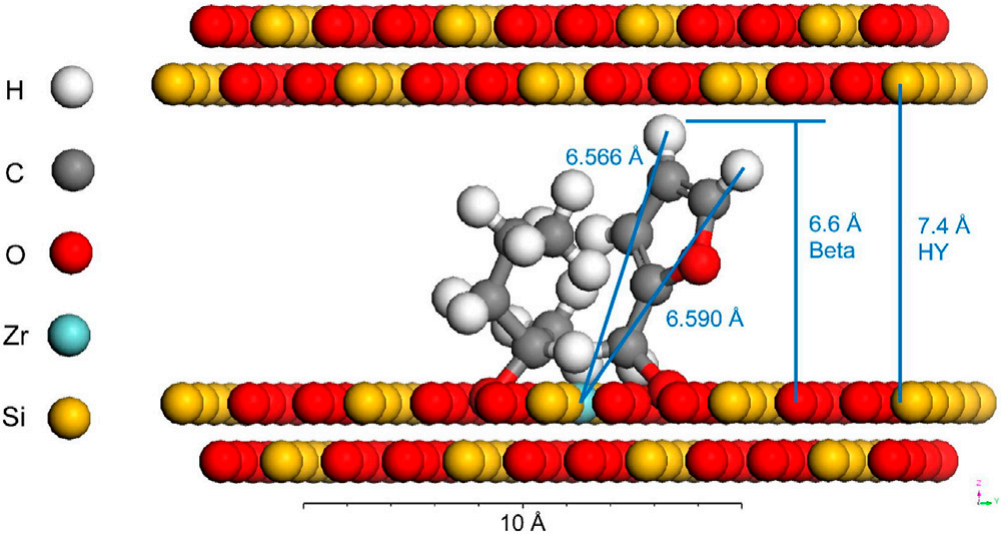
The model of the spatial limitation of the coordination of the catalyst and the substrate in the pore channel [copyright from Zhang et al. (2019a)].

TUD-1 mixed with Zr/Al and zeolite β-type silicate catalyst was prepared and used for a series of conversion of FF (entry 4 and 5 of Table 3) (Antunes et al., 2016). Using 2-BuOH as an H-donor, a stepwise kinetic modeling study was successfully carried out. The effection of catalysts materials on intermediates selectivity was revealed. It was found that the Zr site played a decisive role in the reduction of FF to FA and levulinate (LE) to GVL. It was proved that the coexistence of Al sites can promote the acid catalysis step, involving the process of FA to alkyl furfuryl ethers (FEs), LEs, angelica lactones (AnLs), and LA, respectively. A similar combination also used the catalysts of Zr-HY (Lewis acid) and Al-HY (Brønsted acid) (Zhang et al. (2019a). The Zr-containing zeolite is prepared by a post-synthesis method (entry 6 of Table 3). Zr was added after the dealumination of the parent Al-containing zeolite. However, the model shown in Figure 2 exhibits that there is a certain dimensional limitation in the coordination of FF and 2-PrOH at the Zr Lewis acidic site in the pore channel. Besides, active sites of Zr-Al-Beta zeolites can be changed by mild alkaline treatment (e.g., LiOH, NaOH, KOH) (Gao et al., 2020). It is worth mentioning that alkaline treatment can improve recalcitrance to deactivation and coking, which is beneficial for the recycling of the catalyst.

For the CTH conversion of FF to FA. 2-PrOH as the H-donor over cation-exchanged Lewis acidic BEA zeolite was calculated by DFT (Prasertsab et al., 2018). The catalytic activity of tetravalent metal centers (Sn, Zr, and Hf) substituted into BEA. It found that in the order Zr ≥ Hf > Sn, based on Ea.

In short, the binding capacity of the Hf-zeolite molecular sieve was better than that of the Zr-zeolite molecular sieve. This is because the activation energy of the Zr-zeolite reaction was lower when the Hf-zeolite molecular sieve catalyzed the reaction (Injongkol et al., 2017). The Zr-zeolite catalyst played an important role in the combination of stone and Zr/Hf to form the Lewis acid center, which was believed to constitute the active site of the catalyst. Moreover, it was further determined that this would play an important role in the adsorption and activation of the reactants. The Lewis acid site combined with the carbonyl group to activate the unsaturated C=O double bond, and finally formed the alcohol compound product.

## Zr/Hf-Containing Metal-Organic Framework Catalysts

The term MOF was introduced in 1995 and was now widely accepted (Furukawa et al., 2013). It was a kind of coordination polymer material with good crystal form (Wang et al., 2016a; Goetjen et al., 2020). MOF catalysts have been developed successfully in the application of renewable energy and environmental fields, which have good design capabilities (Zhang et al., 2017; Lin et al., 2021; Mautschke and Llabres, 2021). Highly adjustable porosity and specific surface area, high density of accessible metal sites were the fascinating part of this material (Rui et al., 2021). The microporous zeolite material has diffusion limitations. But the MOF has a mesopore with a diameter of about 9.8 nm, facilitating the full diffusion and mixing of the various substances in the reaction. In terms of BET surface area, zeolite is about 200–500 m^2^ g^−1^, while MOF is about 1,000–10000 m^2^ g^−1^ (Dhakshinamoorthy et al., 2011). Generally, the surface area and porosity of MOF were much higher than those of zeolite, but the chemical and thermal stability of MOFs are not as good as that of zeolite. Zr/Hf-containing MOFs catalysts can be seen everywhere in the field of the transfer hydrogenation of biomass-based carbonyl compounds. Table 4 summarizes the reaction conditions and yields of some excellent Zr/Hf-containing MOF catalysts for the conversion of biomass-based chemicals.

**TABLE 4:** Zr/Hf-containing MOF catalysts.

Entry	Substrate	Catalyst	Condition	H-donor	Product	Conv. (%)	Yield (%)	References
1	LA	ZrF-MOF	200°C, 2 h	2-PrOH	GVL	98	96	Yun et al. (2019)
2	LA	HPW@MOF-808	160°C, 6 h	2-PrOH	GVL	99	87	Li et al. (2021a)
3	ML	UiO-66-S60	140°C, 9 h	2-BuOH	GVL	98	80	Kuwahara et al. (2016)
4	EL	UiO-66(Zr)	200°C, 2 h	2-PrOH	GVL	100	92.7	Valekar et al. (2016)
5	EL	Hf-MOF	120°C, 8 h	2-PrOH	GVL	99	94	Rojas-Buzo et al. (2018)
6	FF	DUT-67(Hf)	180°C, 24 h	2-PrOH	GVL	100	87.1	Li et al. (2019c)
7	FF	M-MOF-808	30°C, 24 h	2-PrOH	FA	94.2	90	Valekar et al. (2020)
8	FF	P/Zr-MOFs	200°C, 2 h	2-PrOH	FA	96.1	96	Wang Y. et al. (2020)

### LA and its Esters as Substrates

For the reaction of LA to GVL, Yun et al. avoided most of the toxic solvent dimethylformamide (DMF) which were typically used in the preparation of Zr polysulfide catalysts (Yun et al., 2019). Instead, they prepared the catalyst ZrF with fumaric acid and ZrCl_4_ in water. In particular, they also used monocarboxylic acids as regulators, which affected the formation and morphology of aquatic zirconia, but did not affect its crystal structure and acidity. ZrF was adjusted with formic acid, acetic acid, and propionic acid, obtaining F-ZrF, A-ZrF, and P-ZrF, respectively. As a comparison, W-ZrF synthesized directly in water without a regulator and D-ZrF prepared in DMF in a traditional mode were also established. The order of particle size is F-ZrF (270 nm) >A-ZrF (130 nm) >P-ZrF (55 nm) >D-ZrF (25 nm). The size of ZrF was related to the molecular weight of the monocarboxylic acid. A regulator with a relatively low molecular weight can obtain ZrF with larger particle size and less excessive aggregation, which is beneficial for catalysis. W-ZrF without modifier did not have a good granular shape but existed in the form of a large block. Among them, at 200°C in 2 h, ZrF prepared by formic acid showed a 98% conversion of LA and 96% yield of GVL (entry 1 of Table 4).

HPW@MOF-808 was fabricated via a facile impregnation method with H_3_PW_12_O_40_ and Zr-based MOF (Li et al., 2021a). Although MOF-808 had good activity in CTH reaction, its Brønsted acidity did not meet the requirements. Therefore, adding HPW (strong Brønsted acid) can effectively promote the esterification of LA with 2-PrOH and the subsequent lactonization reaction. Besides, MOF-808 had high acid resistance, which can exist stably in HCl and its cavity was large enough to encapsulate HPW. The yield of GVL can be obtained by reacting at 160°C for 6 h (entry 2 of Table 4)., and the yield can be increased by 5% when the temperature was increased by 20°C. The dipping method for HPW@MOF-808 was relatively simple, but the preparation of MOF-808 was nearly a week, the process was complicated and time-consuming.

**SCHEME 5:**
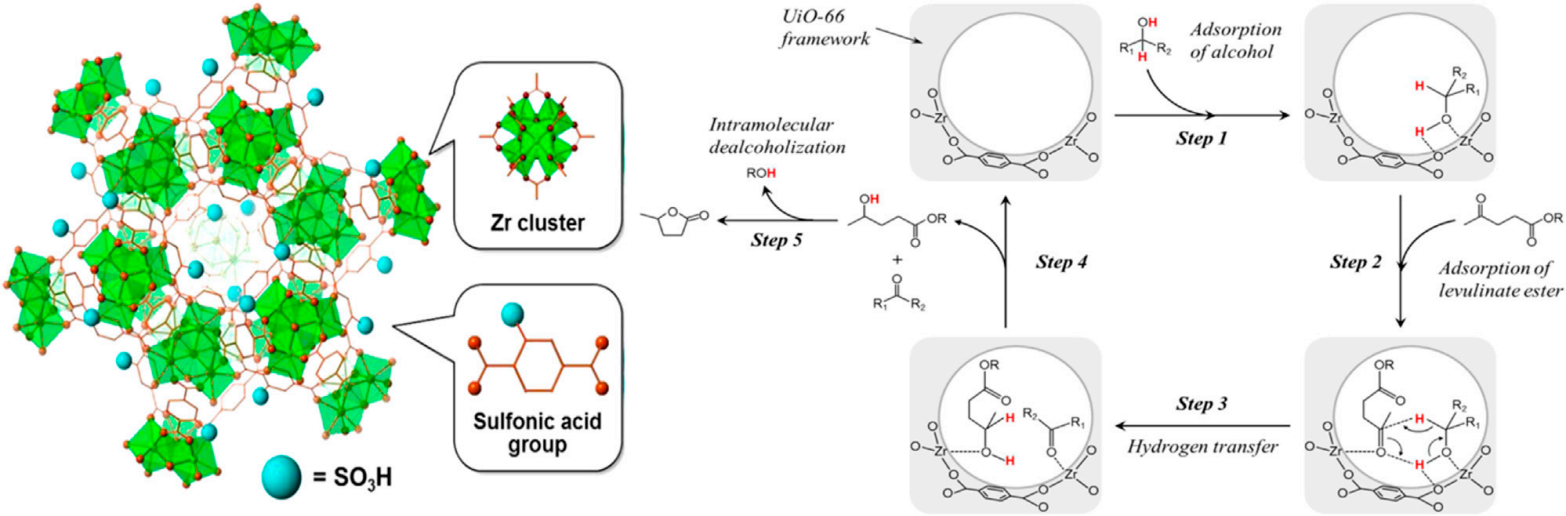
Possible reaction mechanism for CTH process of levulinate esters to produce GVL over UiO-66-S_x_ catalyst and its structure diagram [Copyright from Kuwahara et al. (2016)].

Expanding the starting material from LA to its ester, MOF material catalyst (e.g., sulfonic acid-functionalized UiO-66) also performed well (entry 3 of Table 4) (Kuwahara et al., 2016). UiO-66(Zr) is comprised of 12-coordinated Zr_6_O_4_(OH)_4_ clusters connected with 1,4-benzene dicarboxylate (BDC) linkers (Hu et al., 2016). Due to the very strong force between Zr-O, UiO-66(Zr) material has good water and thermal stability, and the structure can be maintained stably for a long time in a solution of pH = 1–11 (Shearer et al., 2014). Observing Scheme 5, it can be seen that each Zr-O cluster is 12-fold connected to adjacent clusters through a benzene dicarboxylate linker, and stacked together (Kuwahara et al., 2016). Correspondingly, it was confirmed that by FT-IR analysis, the Zr-O cluster only coordinated with the carboxylic acid oxygen atom, but not the sulfonated oxygen atom. With the increased degree of substitution of the sulfonic acid ligand, the XRD intensity and N_2_ adsorption capacity of the microporous solid decreased significantly. In other words, the crystallinity and surface area of MOF materials decreased with the increase of sulfonic acid ligands, defects or irregular connections, and catalyst activity increased. Lewis basic sites (Zr_6_O_4_(OH)_4_ cluster) catalyzed the CTH reaction of LA and its esters (entry 3 of Table 4). Brønsted-acid site (-SO_3_H) promoted continuous intramolecular dealcoholization. The acid-base sites were arranged adjacent to each other in the confined nanospace, and had a synergistic effect on the entire reaction.

A variety of Zr-containing MOFs with different ligands were prepared for the conversion of EL to GVL (Valekar et al., 2016). UiO-66(Zr) showed good catalytic activity (92.7% GVL yield) at a high temperature (200°C), as shown in entry 4 of Table 4. In contrast, MOF-808 has been shown to produce GVL (85% yield) quickly at a moderate reaction temperature (130°C). It is worth mentioning that the latter also performed well in open systems using solvent reflux. In addition, through acid-base modification of the BDC ligand, the changes in its structure and catalytic effect were studied. It was found that the -COOH functional group can provide additional acidity, but the surface area and pore volume of the material have a considerable loss, and the presence of the -NH_2_ group in the ligand provided additional alkalinity to the material, and the surface area loss was small. This was due to the smaller size of the -NH_2_ group compared to the -COOH group. The order of pore size and EL conversion and GVL yield showed consistency, that was UiO-66(Zr)-COOH (25% EL conversion, 13.9% GVL yield) < UiO-66(Zr)-NH_2_ (99% EL conversion, 64.6% IPL yield) < UiO-66(Zr) (100% EL conversion, 92.7% GVL yield). The low catalytic efficiency of UiO-66 (Zr)-COOH was because EL only reacted on its surface or diffused slowly into its narrow pores. On the contrary, when UiO-66(Zr)-NH_2_ was used, almost all EL was converted, but the main by-product IPL was produced. This proved that UiO-66(Zr) functionalized with -NH_2_ will preferentially undergo transesterification with excess 2-PrOH. Similar conversion and yield have been obtained in other work using the same substrate (entry 4 of Table 4) (Rojas-Buzo et al., 2018).

Using EL as the starting substrate, it can be directly converted to GVL through transfer hydrogenation. Sergio et al. prepared a series of materials such as UiO-66 and MOF-808, using formic acid as a modulator (Rojas-Buzo et al., 2018). The latter can be converted into GVL to obtain 100% FF conversion and 97% yield (2 h, 100°C), as shown in entry 5 of Table 4. Compared to UiO-66 (pore size 6Å), MOF-808 has a wider pore structure (pore size 14 Å), which allows the conversion of larger substrates (Mautschke and Llabres, 2021). In the cascade reaction, the combination of Hf-MOF-808 with Al-Beta catalyst was used, and a 51% yield was obtained after 6 h of reaction (entry 5 of Table 4). Hf-MOF-808 showed excellent activity and specific selectivity in hydrogenating carbonyl compounds through the CTH strategy because of its poor crystallinity, defects, large specific surface, and abundant Lewis acid-base sites. By using DFT calculations on the mechanism of MPV reaction, Lin et al. found that carbonyl compounds (e.g., ketones and alcohols) filled the defects of Hf-MOF (Lin et al., 2021). Lewis acid with Hf as the center can coordinate with the oxygen of the substrate molecule, and form a six-membered ring transition state. But other reactive groups with insufficient hardness or large steric hindrance (e.g., -NO_2_, C=C, -CN) were difficult to coordinate with Hf, which was difficult to play a catalytic role. This also explained why Hf-MOF materials have specific selectivity for reducing carbonyl groups.

### FF as a Substrate

For the reaction of FF to GVL, DUT-67 (Hf) performed well. All Hf^4+^ contained in the Hf_6_O_8_ cluster of 12-linked UiO-66(Hf) was saturated, so it is difficult to introduce SO_4_^2-^ through a post-synthetic modification method (Li et al., 2019c). In contrast, each Hf cluster of DUT-67 (Hf) is 8 times connected to the 2,5-thiophenedicarboxylic acid (H_2_TDC) linker (Bon et al., 2013). An inorganic acid (H_2_SO_4_) can be added to introduce Brønsted acid sites. The optimal ratio of sulfated DUT-67(Hf) can convert 100% of FF and undergo a cascade reaction (180°C for 20 h) to finally obtain 84.9% GVL (entry 6 of Table 4). However, the preparation process of the catalyst was very complicated and time-consuming, which was a challenge for the promotion of large-scale use.

In the classic reaction of FF to FA under mild conditions, three Zr-molybdenum compounds (UiO-66, UiO-67, and DUT-52) with the same ligand-metal node coordination but different porous properties were compared. It was proved that the metal node connectivity was more important than high porosity when the Zr-porous membrane material underwent a CTH reaction (Valekar et al., 2020). In addition, the synthesis of an M-MOF-808 material has also been optimized, which can also obtain a 94.2% FF conversion and 90% FA yield after reacting at 40°C for 24 h (entry 7 of Table 4). Low-temperature conditions provided novel ideas for new applications of low-grade waste heat. P/Zr-MOF catalysts were prepared by (NH_4_)_2_HPO_4_ pyrolysis approach starting from Zr-MOFs (Wang Y. et al., 2020). P/Zr-MOFs compared with Zr-MOFs, a part of O–Zr–O in the MOFs was phosphated to form O–Zr–P and P–Zr–P, in which Zr–P possessed stronger Lewis acidity and basicity than those of Zr–O. The increase of reaction temperature and time was conducive to the CTH of FF. However, too high a reaction temperature will result in a decrease in the conversion of FF. Meanwhile, too long reaction time will lead to side reactions of excessive hydrogenation and hydrogenolysis, resulting in a decrease in the selectivity of FA (entry 8 of Table 4).

**SCHEME 6:**
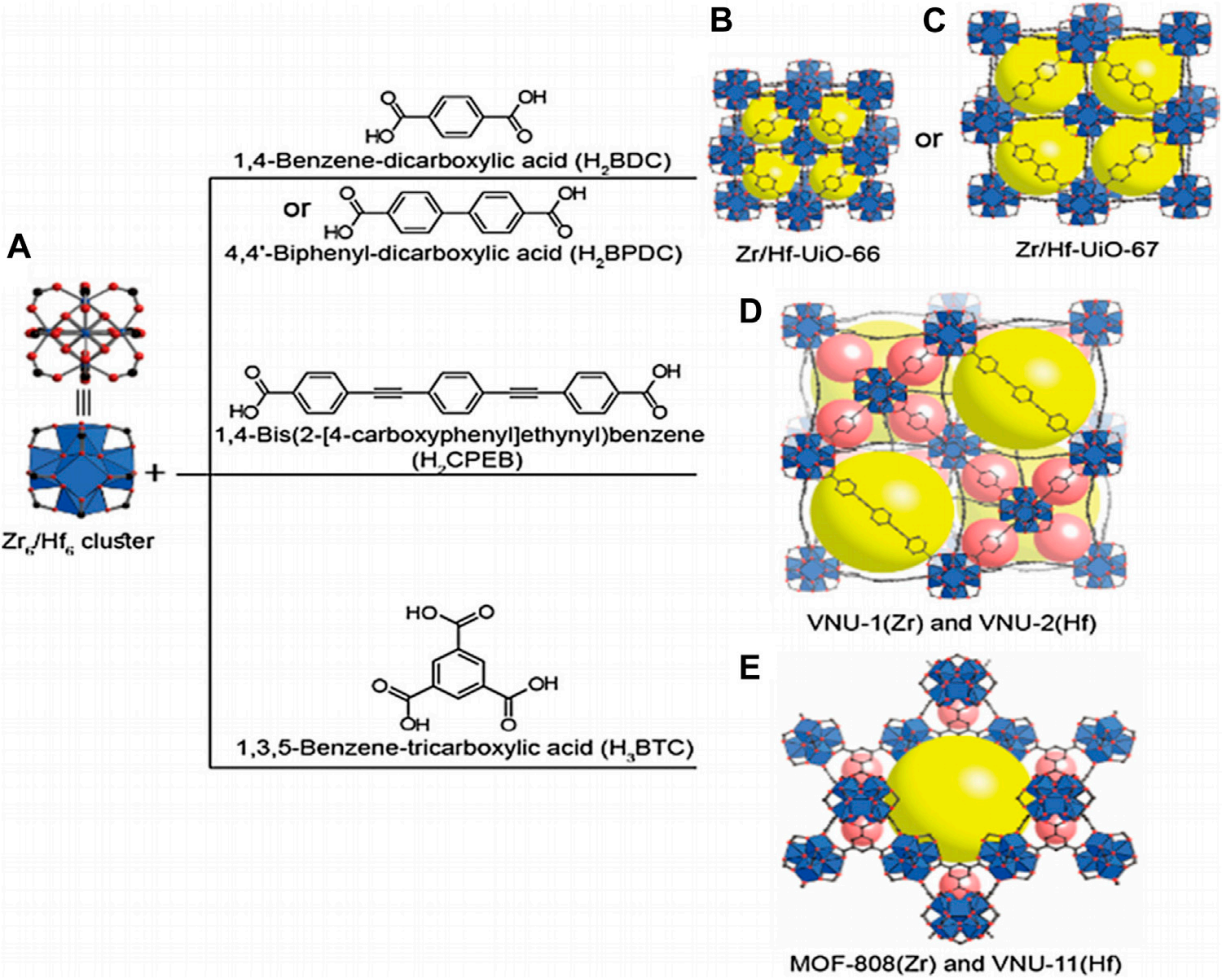
**(A)**. Zr_6_/Hf_6_ cluster. UiO-type MOF structures of **(B)** Zr/Hf-UiO-66 and **(C)** Zr-, Hf-UiO-67. Interpenetrated structure **(D)** of VNU-1(Zr) and VNU-2(Hf). **(E)** Structures of MOF-808 (Zr) and VNU-11(Hf). Color code: C, black; O, red; Zr or Hf, blue; cages, yellow and purple [Copyright from Nguyen et al. (2019)].

In a word, Zr/Hf-containing MOFs exhibited good chemical, thermal, and mechanical stability due to the strong bond between Zr/Hf and oxygen and their high coordination number. Scheme 6 clearly shows the proposed structure of MOFs built from 12-connected and 6-connected Zr_6_/Hf_6_ clusters coordinated with ditopic and tritopic carboxylate linkers, respectively (Nguyen et al., 2019).

## Zr/Hf-Containing Metal-Organic Hydride Catalysts

Metal-organic hydride materials have also been known as unconventional MOF (UMOF) (Gagnon et al., 2012; Goetjen et al., 2020). Compared with the MOF material with good crystal form, Metal-organic hydrides are polymer with an amorphous structure but have good stability and more accessible sites. The organometallic coordination polymerization catalyst has high catalytic activity and good molecular tailoring (Wang et al., 2016b; Liu et al., 2021e). By adjusting the microstructure of catalysts, such as the substituents of the ligand, the coordination atom, and the electronic and three-dimensional environment of the coordination center, the molecular design and assembly of the polymer can be realized at the molecular level (Erickson et al., 2015; Hu et al., 2018; Liu et al., 2019; Peng et al., 2021). In this way, the physical properties of polymers can be controlled, and various polymers with novel functions and stereoisomers can be obtained (Asghari and Yoshida, 2006; Dutta et al., 2012; Jain et al., 2015; Zhou et al., 2019a). Table 5 summarizes the reaction conditions and yields of some excellent Zr/Hf-containing metal-organic hydride catalysts for the conversion of biomass-based chemicals.

**TABLE 5:** Zr/Hf-containing metal-organic hydride catalysts.

Entry	Substrate	Catalyst	Condition	H- donor	Product	Conv. (%)	Yield (%)	References
1	LA	Hf-DTMP	140°C, 3 h	2-BuOH	GVL	98.7	96.9	Hu L. et al. (2019)
2	LA	Zr-BDB	130°C, 3 h	2-PrOH	GVL	99.7	97.2	Song et al. (2021)
3	ML	Zr-BDB	130°C, 4 h	2-PrOH	GVL	99.7	98.4	Song et al. (2021)
4	EL	Zr-BDB	130°C, 6 h	2-PrOH	GVL	99.5	98.7	Song et al. (2021)
5	EL	PPOA-Hf	160°C, 6 h	2-PrOH	GVL	100	85	Wu W. et al. (2018)
6	EL	HA-Zr	150°C, 24 h	2-PrOH	GVL	100	88.3	Xiao et al. (2017)
7	EL	Zr-SRf	150°C, 7 h	2-PrOH	GVL	92.4	92	Zhang et al. (2018)
8	EL	Zr-PhyA	200°C, 1 h	2-PrOH	GVL	100	98.5	Song et al. (2015b)
9	EL	Hf-ATMP	150°C, 4 h	2-PrOH	GVL	95	86	Xie et al. (2016)
10	EL	Zr–CA	150°C, 4 h	2-PrOH	GVL	100	96.9	Xue et al. (2016)
11	EL	FDCA-Hf	160°C, 4 h	2-PrOH	GVL	100	98	Li et al. (2018b)
12	EL	Zr-TMPA	160 °C, 8 h	2-PrOH	GVL	100	96.2	Xie et al. (2017)
13	EL	Zr-HBA	150°C, 4 h	2-PrOH	GVL	100	94.4	Song et al. (2015a)
14	EL	Hf-OFR	150°C, 9 h	2-PrOH	GVL	91	86	Chen et al. (2021a)
15	EL	Hf-GO	150°C, 5 h	2-PrOH	GVL	95.5	87.7	Li et al. (2020c)
16	EL	Hf-GO	150°C, 5 h	2-PrOH	GVL	54.8	54.7	Li et al. (2020c)
17	EL	Hf-DTMP	140°C, 3 h	2-BuOH	GVL	97.63	96.2	Hu L. et al. (2019)
18	BL	Zr-BDB	130°C, 6 h	2-PrOH	GVL	81.4	77.2	Song et al. (2021)
19	BL	ZrPO-1.00	210°C, 2 h	2-PrOH	GVL	98.1	95.7	Li et al. (2017)
20	FF	ZrPN	140°C, 2 h	2-PrOH	FA	98	98	Li et al. (2016c)
21	FF	Zr-LS	100°C, 1 h	2-PrOH	FA	97.5	96	Zhou et al. (2019b)
22	FF	Zr-LS	80°C, 3 h	2-PrOH	FA	99	90	Zhou et al. (2019b)
23	FF	PhP-Hf	120°C, 2 h	2-PrOH	FA	99.2	97.6	Li et al. (2019a)
24	FF	Zr-HAs	50°C, 15 h	2-PrOH	FA	97.4	96.9	Sha et al. (2017)
25	FF	Hf–TA	70°C, 3 h	2-PrOH	FA	100	99	Wang X. et al. (2020)
26	FF	Hf-DTMP	130°C, 3 h	2-BuOH	FA	99.9	98.5	Hu et al. (2019b)
27	FF	HPW/Zr-β	160°C, 24 h	2-PrOH	GVL	100	68	Winoto et al. (2019)
28	HMF	Hf-DTMP	100°C, 2 h	2-BuOH	BMHF	99	90	Hu et al. (2019b)
29	HMF	MZCCP	130°C, 4 h	2-BuOH	DHMF	83.9	64.2	Hu et al. (2018)

### LA and its Esters as Substrates

Using LA as a substrate, a new type of organic zirconium borate (Zr-BDB) was developed by Song et al. to convert it to GVL (Song et al., 2021). 1,4-Benzodiboric acid (BDB) and ZrOCl_2_.8H_2_O were synthesized by a solvothermal method in DMF (entry 1 of Table 5). As a Lewis acid, BDB can coordinate with and activate the hydroxyl group in alcohol. At the same time, Zr was combined to activate the carbon group, achieving the purpose of synergistically catalyzing the MPV reaction. The yields of different substrates (ML > EL > BL) to GVL were due to the increased steric hindrance from methyl to butyl.

In the system of EL to GVL, there were many different catalysts, such as Zr-BDB, PPOA-Hf, HA-Zr, Zr-SRf, Zr-PhyA, Hf-ATMP, Zr–CA, 2,5-furandicarboxylic acid (FDCA)-Hf, Zr-TMPA, Zr-HBA, Hf-OFR, Hf-GO, Hf-DTMP (entry 4–17 of Table 5). All of them were coordinated by different organic ligands. Hf-OFR was prepared by the oxytetracycline fermentation broth residue (OFR), which is an abundant solid waste in the fermentation industry (Chen et al., 2021a). It is beneficial for both pharmaceutical wastes and bio-based resources. Hazardous and tricky to treat. EL was completely converted to obtain GVL with a yield of 85% (160°C, 6 h) by PPOA-Hf catalyst (Wu et al., 2018b). Ea was estimated at approximately 53 kJ/mol, which was relatively lower than other catalysts. The layered metal phosphonate-phosphate hybrid can be used for ion exchange. Using humic acid (HAs) as a raw material to synthesize Zr-containing catalyst (Zr-HA) was proposed (Xiao et al., 2017). HAs are an important part of lignite (typical low-rank coal) with abundant reserves. This low-rank coal is usually only used as a low-quality fuel. If it can be applied to fine chemical catalysts, it will be a new method to realize the classification and clean utilization of coal. Similarly, HA and the solid residues (SR) after HA extraction from lignite were used to prepare Zr-SRf catalyst (Zhang et al., 2018). Compared to the previous study, this work had greatly shortened the reaction time (7 h V.S. 24 h) and improved the selectivity (83% V.S. 99.5%). Besides, graphene oxide (GO) is rich in acidic carboxyl groups and phenolic hydroxyl groups (Palermo et al., 2016; Karthik et al., 2021). Through the coordination of Hf^4+^/Zr^4+^ with the carboxyl group in graphene oxide, Hf-GO, and Zr-GO catalysts were constructed (Li et al., 2020c).

There are not many studies on BL as the substrate, and zirconium phosphates were used in this reaction (Li et al., 2017). The BL conversion at 2 h was 98.1%, and the GVL yield was 95.7% (entry 19 of Table 5). Compared with ZrO_2_, the binding energies of Zr3d and O1s of all ZrPO-X materials were significantly increased. This was due to the introduction of phosphorus, leading to the decrease of Zr electron density and the increase of surface hydroxyl groups.

### FF as a Substrate

For the conversion of FF to FA, a template-free method to synthesize various heterogeneous acid−base bifunctional nitrogen-containing alkyltriphosphonate-metal hybrids (MPN) catalysts were synthesized by Li et al. (Li et al., 2016c). The organotriphosphate-zirconium hybrid (ZrPN) mainly focused on the CTH reactions of biomass-derived carbonyl compounds to alcohols and aldoses to ketoses (entry 20 of Table 5). Through calculation, the rate constant of ZrPN at 100°C was 3.6 × 10^−3^s^−1^, which was 12 times than that of ZrO_2_. The Ea of ZrPN and ZrO_2_ were 70.5 and 79.1 kJ/mol, respectively. Both two proved the superior performance of the Zr N-Alkyltriphosphate nanohybrid from the perspective of reaction kinetics. It’s worth paying high attention that the intramolecular CTH of C3−C6 aldoses to the corresponding ketoses in water was also studied. Generally, Al-zeolites can not catalyze glucose isomerization due to the weakening of Lewis acid sites existing in water. In contrast, Lewis acid sites in ZrPN can not be hampered by water, which showed excellent potential in CTH reaction. In another word, ZrPN can be extrapolated to other similar lignocellulose-derived substrates for upgrading biomass.

The conversion of FF can also be achieved by simply assembling lignosulfonate and ZrCl_4_ to form a Zr-containing polyphenol biopolymer catalyst under hydrothermal conditions (entry 22 of Table 5) (Zhou et al., 2019b). Lignosulfonate has a sulfonic acid group and can be used as an alkali-free acidic site. Therefore, dangerous sulfonation can be avoided. Catalytic experiments were also carried out with 18 typical aldehydes and ketones in the traditional chemical industry as substrates. It proved that Lignosulfonate-based catalysts had good universality and application potential. Different proportions of phenylphosphonic acid (PhP) and Hf were used to prepare the acid-base bifunctional nano-hybrid catalyst PhP-Hf, which can convert FF to FA (entry 23 of Table 5) (Li et al., 2019a). The catalyst had a moderate acid-base site and acidity. PhP-Hf was weaker than that of HfO_2_, but it also promoted the reaction. Acid (Hf^4+^) and base (O^2-^) sites had a synergistic effect on the CTH reaction of FF to FA. The base sites facilitated the adsorption and dissociation of 2-PrOH on the catalyst, and the aldehyde group was activated by Hf^4+^. Then the six-membered ring transition state was formed *in situ* and completed the hydrogen transfer. And the Ea was calculated at about 60.8 kJ/mol.

**SCHEME 7:**
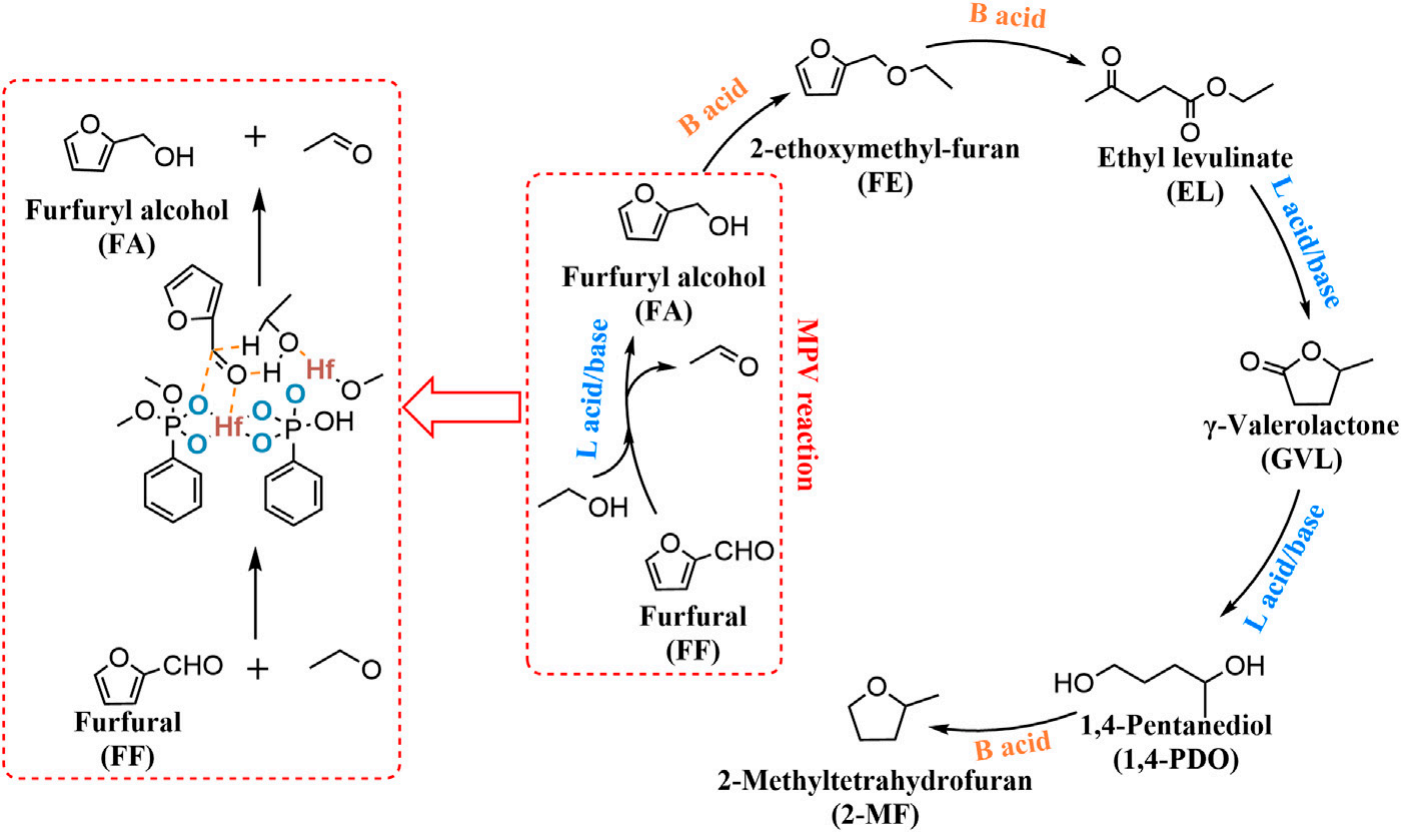
Mechanism of FF to GVL over PhP-Hf.

The Hf-TA catalyst was synthesized in DMF with naturally occurring tannic acid (TA) as a ligand to coordinate with HfCl_4_. (Wang et al., 2020d). The advantage of this method was that renewable and natural resources were used to construct an efficient and environmentally friendly Hf-containing catalyst, and the preparation process was simple. Although the general catalyst can only be recycled about 5 times, the catalytic effectiveness of the 10th used catalyst was better than the first time. It was further confirmed by FT-IR spectra, showing that the residual DMF in the pore structure of the catalyst was gradually replaced by 2-PrOH during the reuse process. This would facilitate contact with more active sites, increasing the MPV activity. Under the relatively mild reaction conditions (70°C, 3 h), a high yield of 99.0% of FA can be obtained (entry 25 of Table 5). Zirconium phosphonate The materials synthesized by the coordination of organic acids and zirconium were widely used in the biomass-based conversion. Zr-containing catalysts such as Zr-HAs, Zr-DTMP, HPW/Zr-β (entries 24, 26, 27 of Table 5) also can be used in similar systems from FF to FA or GVL. By regulating the ratio of organic phosphonate and inorganic phosphate with the center of Zr metal (Silbernagel et al., 2016), further optimization of the MPV reduction reaction system could be successfully carried out. The specific active sites required for each step of FF to GVL were shown in Scheme 7. Lewis acid/base sites were responsible for the MPV reduction (FF to FA, and EL to GVL), while Brønsted acid sites exhibited key effects on the etherification reaction (FA to FE) and ring-opening reaction (FE to EL).

### HMF as a Substrate

Converting HMF to BHMF is also a significant path for biomass-derived conversion. A new Hf-based metal-organic coordination polymer (Hf-DTMP) was assembled by HfCl_4_ and diethylene triaminepenta (methylene phosphonic acid) (DTMP) (Hu et al., 2019b). The mechanism was that 2-BuOH, and HMF were first adsorbed on catalyst. Subsequently, 2-BuOH was dissociated into the active H and alkoxide by O^2-^. Meanwhile, the carbonyl group of HMF was activated by Hf^4+^. Then, a six-membered intermediate was generated by the dissociated alcohol and carbonyl group with Hf^4+^-O^2-^. Finally, BHMF was eventually formed *via* CTH (entry 28 of Table 5). In contrast, the magnetic catalysts (MZCCP) synthesized by ZrCl_4_, cyanuric acid (CA), and Fe_3_O_4_ did not have an advantage in terms of yield (90% V.S. 64.2%) (entry 29 of Table 5) (Hu et al., 2018).

## Conclusion and Perspectives

Mountains of researches proved that CTH is a significant strategy to transform biomass-based substrates into chemical platform products and fuel precursors of high value. Zr/Hf can be supported on other materials or coordinated/coupled with them to form Zr/Hf-containing materials, of which they can play a significant role in CTH that is similar to noble metal catalysis. In this review, the catalytic effects and mechanisms of different types of Zr/Hf-containing catalysts are discussed. Various kinds of materials demonstrate different properties and preparation methods. Zr/Hf-containing oxides are relatively inexpensive and easily available. However, their basic sites are not strong and are easily affected by solvents, and the corresponding reaction activity is not ideal. Supported catalysts containing Zr/Hf can use existing templates to enhance stability and increase surface area. The zeolite catalysts are prone to be affected by the hydroxyl groups on Si, and the acidic enhancement of Lewis can effectively fix the metal and coordinate. The MOF catalysts generally have a good crystal form and spatial structure. However, the preparation process of these three catalysts is complicated and the relevant precursors are expensive, which is not suitable to apply from an economic point of view. Relatively speaking, the preparation steps of metal-organic hybrid materials are simpler and the time is shorter, which have abundant sources and room for expansion.

To establish a mature industrialization system, economic benefits and environmental impacts should be taken into account. In other words, the preparation and reaction process of the catalyst should be as simple and time-saving as possible. Also, the consumption of raw materials and energy should be low. Correspondingly, the purity and quality of the obtained product should be high. The problem at this stage is that there are seasonal differences in the availability of biomass raw materials, and the competitiveness of economic benefits is not very strong. As the saying goes, there is no useless waste, only misplaced resources. How to convert this huge amount of useless waste into resources that can replace the heavily polluting traditional energy and generate economic benefits is a problem that needs to be tackled on the road to green development. And this depends on the joint efforts of scientific researchers, enterprises, and the government, respectively. We still need to propose a new solid acid catalyst design strategy. 1) Using non-precious metal materials and green solvents to achieve a win-win situation for the economy and environmental protection. 2) Increasing the density of acid and base sites accessible to the catalyst. 3) Controlling the ratio of Lewis/Brønsted acid sites. 4) Developing more functional group synergistic catalysis. 5) Modulating the wettability of the catalyst surface together with increasing its water resistance. 6) Simplifying the catalyst preparation process, reducing the preparation cost, and controlling the energy consumption of each link accurately.
